# Improvement of the Uranium Sequestration Ability of a *Chlamydomonas* sp. (ChlSP Strain) Isolated From Extreme Uranium Mine Tailings Through Selection for Potential Bioremediation Application

**DOI:** 10.3389/fmicb.2018.00523

**Published:** 2018-03-21

**Authors:** Beatriz Baselga-Cervera, Julia Romero-López, Camino García-Balboa, Eduardo Costas, Victoria López-Rodas

**Affiliations:** Department of Animal Production (Genetics), School of Veterinary Medicine, Universidad Complutense de Madrid, Madrid, Spain

**Keywords:** *Chlamydomonas*, extremotolerant, anthropogenic extreme environments, uranium tailings, artificial selection, uranium sequestration, bioremediation

## Abstract

The extraction and processing of uranium (U) have polluted large areas worldwide, rendering anthropogenic extreme environments inhospitable to most species. Noticeably, these sites are of great interest for taxonomical and applied bioprospection of extremotolerant species successfully adapted to U tailings contamination. As an example, in this work we have studied a microalgae species that inhabits extreme U tailings ponds at the Saelices mining site (Salamanca, Spain), characterized as acidic (pH between 3 and 4), radioactive (around 4 μSv h^−1^) and contaminated with metals, mainly U (from 25 to 48 mg L^−1^) and zinc (from 17 to 87 mg L^−1^). After isolation of the extremotolerant ChlSP strain, morphological characterization and internal transcribed spacer (ITS)-5.8S gene sequences placed it in the *Chlamydomonadaceae*, but BLAST analyses identity values, against the nucleotide datasets at the NCBI database, were very low (<92%). We subjected the ChlSP strain to an artificial selection protocol to increase the U uptake and investigated its response to selection. The ancestral strain ChlSP showed a U-uptake capacity of ≈4.30 mg U g^−1^ of dry biomass (DB). However, the artificially selected strain ChlSG was able to take up a total of ≈6.34 mg U g^−1^ DB, close to the theoretical maximum response (≈7.9 mg U g^−1^ DB). The selected ChlSG strain showed two possible U-uptake mechanisms: the greatest proportion by biosorption onto cell walls (ca. 90%), and only a very small quantity, ~0.46 mg g^−1^ DB, irreversibly bound by bioaccumulation. Additionally, the kinetics of the U-uptake process were characterized during a microalgae growth curve; ChlSG cells removed close to 4 mg L^−1^ of U in 24 days. These findings open up promising prospects for sustainable management of U tailings waters based on newly evolved extremotolerants and outline the potential of artificial selection in the improvement of desired features in microalgae by experimental adaptation and selection.

## Introduction

Around the globe, U mining and milling operations have taken place in different countries producing large amounts of liquid and solid residues, more than 936 × 10^6^ m^3^ of tailings (Abdelouas, 2007). Both operational and inactive U mines have a negative impact on all adjoining areas both *in situ* and *ex situ* (soils, ground waters, effluents, surrounding biosphere). The radiological and chemical legacies of the exploitation system are the associated tailings that contain hazardous material, including U and other heavy metals, and products of U decay chains (Lozano et al., 2000; Antunes et al., 2007). U is radioactive and one of the heaviest natural chemical elements (Emsley, 2003; Závodská et al., 2008). The toxicity of U as a heavy metal is significant, and is considered a greater hazard than its natural radioactivity (Markich, 2002). The biological effects of U are similar to other heavy metals, and U ionic radiation presents potential teratogenic, mutagenic, and carcinogenic effects (Keith et al., 2013). Owing to the high mobility and solubility of U(VI) stable aqueous complexes in natural water systems (Kim et al., 1992), U could potentially migrate into the environment and may be present in significant amounts in freshwater reservoirs. In areas influenced by U mining activities, it is known to occur at high concentrations, exceeding the World Health Organization drinking-water guidelines limit values at 30 μg L^−1^ (WHO, 2012). For example, up to 700 μg L^−1^ were found in Canada (Moss et al., 1983) or above 1 g L^−1^ in mining areas (Roig et al., 1997; Dessouki et al., 2005; Kalin et al., 2005; Merkel and Hasche-Berger, 2006; Neves and Matias, 2008). Therefore, the Uranium Mill Tailings Remediation Action (UMTRA) project in the USA has established a concentration limit of 0.05 mg L^−1^ (Anderson et al., 2003), and U needs to be removed from aquifers and surface waters in terms of U mine environmental restoration.

Usually, physical and chemical methods have been employed for U mine water remediation (e.g., adsorption, membrane microfiltration, reductive precipitation, ion exchange, reverse osmosis, or flocculation) (Li and Zhang, 2012; Bhalara et al., 2014). Unfortunately, cleaning up contaminated areas with these methods is arduous, often with a high economic cost. Recently, alternative procedures are being increasingly developed, such as advanced protein engineering to selectively take up very low concentrations of U (Zhou et al., 2014) or by classic bioremediation procedures using living and non-living biomass/biological components to recover U with varying success (Suzuki and Banfield, 2004; Kalin et al., 2005; Sousa et al., 2013; Chung et al., 2014). It appears that the use of microbes is the most interesting alternative to treat low concentrations of U affecting large areas (Gavrilescu et al., 2009; Sousa et al., 2013). The high proportion of negatively charged ionic groups in the cell walls makes microalgae very favorable for biosorption of uranyl cations (Horikoshi et al., 1979; Fortin et al., 2004, 2007; Dessouki et al., 2005; Yusan and Akyil, 2008; Acharya et al., 2009; Cecal et al., 2012; Tripathi et al., 2013). In practical terms, for example, the addition of microalgae in a U mine pit-lake caused a 13% decrease in U concentration (Dessouki et al., 2005), and microbial-mediated natural remediation has been used for two flooded U pits in Canada (Quiring, 2008; Muldoon and Schramm, 2009). Most of the experiments to recover U were performed using microalgae strains from culture collections, which had not previously been in contact with U. Thus, little information is available on natural strains adaptive to extremely U-polluted environments and only the natural physiological capacity of these lab strains for U uptake was explored. However, the regular discovery of microbial life in even more harsh environments has changed our basic understanding of the boundaries of life (Glover and Neal, 2012). Microorganisms thriving in U tailings ponds may have developed adaptions to survive and resistance to the conditions imposed, particularly related to U. Such adaptive features represent stock options to the biotech and bioremediation field. Therefore, it is of utmost importance to study the performance of U tailings pond microorganisms and whether it is possible to increase their uptake of U.

One example of these type of places are the former U mining and milling sites in Saelices el Chico, Salamanca, Spain (European Commission, 2012). For more than 20 years, as a result of the extraction and processing of U from the ore-base stable reservoirs, several tons of waste, slag dumps and tailings have been generated and routinely discharged on the field. Recent ecological and toxicological studies have been performed to investigate the status and impact after restoration (García-Balboa et al., 2013; Ramírez-Paredes et al., 2013). Physicochemical and ecological characterization found that acid mine drainages present a high content of heavy metals, and an acute lethal toxic effect for the bacteria *Vibrio fisheri* and two algal strains (García-Balboa et al., 2013; Ramírez-Paredes et al., 2013). Despite the hostile conditions, several bacteria and eukaryotic microorganisms have been described living in the Saelices U tailings ponds (Muñoz et al., 1995; García-Balboa et al., 2013). In previous studies, we have characterized the phytoplankton and chemical profile of the U tailings ponds, the first step toward bioprospection (Baselga-Cervera et al., 2013; García-Balboa et al., 2013). The aim of the present work is to describe the identification and physiology of a *naturally-adapted* microalga that inhabits these environments (i.e., pit water and tailings ponds from a restored U mine), study its bioremediation capacity and test the feasibility of an artificial selection process for microalgae to optimize U uptake. For this purpose, we first identified and characterized the most suitable species for artificial selection (*Chlamydomonas* sp.). In addition, we have subjected the U-resistant *Chlamydomonas* sp. to an artificial selection process to maximize its capacity for U uptake and studied its performance in U sequestration. We also present and discuss different systems for microbial bioremediation of U-polluted waters and the potential of our system.

## Materials and methods

### Study site and sample collection

The Hesperian Massif in the Iberian mainland (Spain) has large amounts of U ore, one of the biggest U deposits in Europe, mineralized as pitchblende and black oxides, filling fractures of schists and slates of the schist-greywacke Cambrian and Pre-Cambrian complex (Marina and Vázquez Guzmán, 1987). Within this geographical area, several Spanish U mining and milling sites, in operation from 1974 to 2000, are currently under environmental monitoring after restoration. The former Saelices mining facility (Saelices el Chico, Ciudad Rodrigo, Salamanca, Spain) has two storage ponds and several open pits, one of them, Saelices pond is an open pit area with a 400,000 m^3^ maximum capacity. Here, a pit lake forms every rainy season due to the storage of water streaming from former restored and non-restored contaminated sectors (waste-dumps, open pits, heap leaching piles, etc.). Annually, 500,000 m^3^ of contaminated water (surface, seepage, mining, groundwater) are collected for treatment (European Commission, 2012). Water samples containing microalgae were taken from tailings ponds and acid waters from open pits at the Saelices mine site, which were extremely contaminated by U (from 25 to 48 mg L^−1^) and other metals, stored in sterile containers and transported to the laboratory [samples were provided by *Empresa Nacional del Uranio* (ENUSA)]. Samples for microalgae isolation were transferred to cell culture flasks (Greiner, Bio-One Inc., Longwood, USA) and preserved for subsequent isolation, for >3 days, at 20 ± 2°C. Mine water samples were filtered through EMD Millipore filters, 0.22 μm pore size (sterile, Fisher Scientific, Madrid, Spain), and stored in darkness at 4°C until used. All the procedures performed with U mine waters made use of the same water, from now on referred to as Saelices water. At the onset of the experimental procedures, pH, conductivity (Crison Instruments, Barcelona, Spain) and total U were quantified in the Saelices water. Those samples for metal analysis were previously acidified with 2% nitric acid. Saelices water was analyzed for U (detection limit 0.1 mg L^−1^) using inductively coupled plasma-mass spectrometry (ICP-MS; VARIAN RedTop ICP mass spectrometer, Palo Alto, USA) and semi-quantitative elements distribution (ThermoFisher iCap Q ICP mass spectrometer, Massachusetts, USA).

### Strain isolation and culturing

U mine water samples were serially diluted up to 10^5^ and single cells were isolated using micropipettes. Strains were continually subcultured in BG-11 medium and re-isolated until pure cultures were obtained and deposited in the Algal Culture Collection of Universidad Complutense de Madrid (Spain). Morphological identification at the genus level was accomplished according to the methods of Bellinger and Sigee (2015) and by comparison with the AlgaeBase database (AlgaeBase Listing the World's Algae, 2015). An inverted phase contrast fluorescence microscope (Axiovert 35, Zeiss, Oberkochen, Germany) with a coupled camera (Axio Cam MRc, Zeiss) was used for morphological analyses and micrographs.

For regular maintenance, strains were grown in 50–250 mL cell culture flasks (Greiner, Bio-One Inc., Longwood, USA) with filtered U mine water or freshwater enriched with BG-11 broth (Sigma-Aldrich, Germany), and maintained at 20 ± 2°C and a constant irradiance of 80 μmol photons m^−2^ s^−1^ over the waveband 400–700 nm from cool white fluorescent tubes. Cultures were maintained under non-synchronized balanced growth, in exponential growth phase (Reynolds, 1992), by serial transfers of a small inoculum to fresh culture medium every 20 days. Cells were monitored by optical microscopy to address motility, shape, viability, and recombination forms. Cellular densities were directly counted under the microscope using a hemocytometer (Hoshaw and Rosowski, 1973). Cultures were regularly checked for possible contamination by patting in nutrient agar and direct observation under a microscope after staining with acridine orange.

The experiments were conducted using one of the mine water samples (the sample referred to as Saelices, water from a large tailings evaporation pond in Saelices mine), except where otherwise indicated. Saelices water filtered (0.22 μm filter Millipore, Merck KGaA, Darmstadt, Germany) and enriched with BG-11 broth was used as culture medium. The pH of Saelices medium, pH ≈3.3, was monitored throughout the entire growth period, up to 48 days, and did not exceed the value of 5. No signs of the formation of precipitates in the Saelices medium and microalgae cultures were observed. The U-uptake test was carried out in BG-11 medium with ^238^U radioisotope (as uranyl nitrate, Sigma-Aldrich, Germany); solutions were prepared in crystal jars, left to equilibrate and adjusted to pH 5 (using 0.1 M KOH). Low pH was selected at the onset of the algae uptake experiment; soluble uranyl ions occur mainly in acidic waters (Závodská et al., 2008).

### Electronic microscopy analysis

Cells of the strain ChlSP were harvested by centrifugation (3,500 rpm and 5 min), fixed with equal parts of fresh 2.5% (v/v) glutaraldehyde and 4% (v/v) purified paraformaldehyde buffered with 0.1 M sodium cacodylate, pH 7, at 4°C overnight, and stained in 2% (v/v) OsO_4_ for 1 h at 4°C. Pelleted cells were dehydrated in increasing concentrations of acetone and embedded in Spurr resin. Sections of 80 nm thickness (sectioned with an LKB 2088 ultra-microtome) were collected on copper grids (Sigma-Aldrich, Germany) and examined with a JEOL JEM-2010 transmission electron microscope (Jeol Ltd., Tokyo, Japan) operated at 100 kV.

### DNA extraction, PCR amplification, and sequencing

Total genomic DNA from the isolated ChlSP strain was extracted following the method of Rivas et al. (2001) with minor modifications. Cells were harvested by centrifugation at 9,000 g in a Microspin centrifuge (Biocen22, Ortoalresa, Madrid, Spain) for 10 min at room temperature and lyophilized for 5 h. DNA was extracted with 100 μL of 0.05 M NaOH (DNA free) by heating at 100°C for 5 min. Samples were then placed in an ice bath and 900 μL of water was added to each microtube and mixed thoroughly. After an additional centrifugation at 9,000 g, 700 μL of the supernatants were harvested and frozen at −20°C until use.

Amplifications of the internal transcribed spacer (ITS) and 5.8S regions of the ribosomal DNA were performed by PCR using the primers: TW81 (5′-GGGATCCGTTTCCGTAGGTGAACCTGC-3′) and AB28 (5′-GGGATCCATATGCTTAAGTTCAGCGGGT-3′) (Goff and Moon, 1993). PCR was performed using *Taq* DNA Polymerase (Amersham Pharmacia Biotech, New York, USA), following the manufacturer's instructions: 25 μL final reaction volume with 5–10 ng of genomic DNA, 2.5 μL of *Taq* polymerase buffer 10×, 1 μL of BSA 0.1%, 2.5 μL of dNTPs mix (2 mM), 2.5 μL of each primer (2 μM), and 1 U of *Taq* DNA polymerase. PCR amplification was carried out on a Whatman Biometra® T-gradient thermocycler (Göttingen, Germany) with an initial preheating at 97°C for 5 min; 37 cycles of denaturing at 95°C for 1.25 min, annealing at 60°C for 2 min and extension at 72°C for 4 min; and a final extension at 72°C for 7 min.

To check the amplification process efficiency, 5 μL of the amplification reactions were analyzed by electrophoresis in TAE buffer in a 1% (w/v) agarose gel and visualized under UV light after staining with ethidium bromide. The bands corresponding to the ITS1, 5.8S, and ITS2 of the rDNA genes were directly purified from the respective agarose gels by centrifugation using the QIAquick® Gel Extraction kit (Qiagen, Hilden, Germany). Sequencing reactions were performed on an ABI377 sequencer (Applied Biosystems, Foster City, USA) using a BigDye Terminator v3.0 cycle sequencing kit with the same primers used for amplification.

### Phylogenetic analyses

The forward and reverse sequences obtained from the two ChlSP clones were assembled, edited and deposited in GenBank. Additional sequences included in the phylogenetic analyses were selected and downloaded from the NCBI database by statistical significance of the matches using BLAST analysis and including a *Chlorella vulgaris* sequence (AY591514.1) for outgroup rooting. The consensus sequences were preliminarily aligned using MUSCLE (Edgar, 2004), visually inspected in MEGA7 (Kumar et al., 2016), and later refined using Gblocks 0.91b (Castresana, 2000) allowing gap positions within final blocks. The final ITS-5.8S alignment was reduced to 382 positions, and analyzed with maximum likelihood (ML) and Bayesian inference (BI), using unlinked GTR+G models with RaxML (Anisimova et al., 2006) and MrBayes (Huelsenbeck and Ronquist, 2001), respectively. Robustness of the ML tree was determined by computing bootstrap percentage (BP) to estimate the individual clade confidence limits from 1,000 resamplings. BI analysis was performed using two parallel Markov chain Monte Carlo runs for 100,000 generations, each with one cold and three heated chains. Trees were sampled every 100 generations and the first 1,000 trees were discharged as burn-in. A 50% majority-rule consensus of the sampled trees was generated containing the Bayesian posterior probabilities (PP) of the three nodes.

### Artificial selection

We maximized the genotypic diversity of the ancestral wild-type ChlSP strain by inducing sexual reproduction using the classic procedure of nitrogen depletion (Sager and Granick, 1954). A dense ChlSP culture was grown under nitrogen depletion until cell division ceased. We observed sexual reproduction in the cultures using a Zeiss III Axiovert inverted microscope (Jena, Germany). First, the cells divided into numerous protoplasts, which then differentiated into motile gametes that were released. Fertilization between isogametes resulted in numerous zygotes. After we added fresh BG-11 medium, meiosis resulted in four zoospores per zygote, and new motile vegetative cells appeared by successive divisions. The new cell population was maintained in exponential growth by serial transfers of an inoculum to fresh culture medium every 20 days.

The artificial selection procedure was based on a three-step procedure (Costas Costas et al., 2012; Baselga-Cervera, 2017). We added (without causing turbulence) a large population of cells originating from divisions of the zoospores that appeared after meiosis (around 10^9^) at the top of a 60 cm long × 11 cm outer diameter crystal burette filled with 25 mL of Saelices medium. The culture was maintained in dark conditions for almost 2 h in order to stimulate a positive phototactic response (*Chlamydomonas* cells have positive phototaxis after 1 h or more under dark conditions; Musgrave and Häder, 1991). The burette was covered with aluminum foil except for the top, which was irradiated with 250 μmol photons m^−2^ s^−1^ over the waveband 400–700 nm to cause the cells to swim toward the light. Then, cells were allowed to swim or settle for 15 min (most cells tended to swim toward the light, but some settled instead). Finally, we selected the ~500 cells that settled fastest for the next generation. We assumed that a faster sinking velocity corresponds to a higher U uptake by the cells, resulting in increased density or/and an increase in the area of the cells (implying more binding sites in the cell walls). The selected cells were propagated in fresh Saelices medium for 20 days until the next selection cycle.

We found a new strain after 12 cycles of selection with the resulting selected cells, under the same laboratory conditions as outlined above. This culture constituted the artificially selected Saelices population (designated as ChlSG strain) that is stored in the Algal Culture Collection of Universidad Complutense de Madrid (Spain), as well as deposited in the Banco Español de Algas (BEA D04-12). Assuming that the capability for U uptake is a quantitative genetic trait, the response to the artificial selection (*R*) can be computed in accordance with (Falconer and Mackay, 1996):

$$R\;=\;\mu_{\text{a}}-\;\mu_{\text{gi}}$$

where μ_a_ and μ_gi_ are the overall mean total U taken up by the ChlSP and the ChlSG strains, respectively.

We also estimated the theoretical maximum response (*R*_max_) (Falconer and Mackay, 1996):

$$R_{\max}\;=ih^{2}\sigma_{p}$$

where *i* is the intensity of selection obtained from the Falconer's nomogram (Falconer and Mackay, 1996), *h*^2^ is the heritability computed as the slope of the regression line between the mean values of the ChlSP and the ChlSG strains, and σ_p_ is the standard deviation of the observed values of U uptake.

### *In vivo* visualization of the U-uptake process

Time-lapse micrographs of *Chlamydomonas* sp. strain ChlSP were obtained using a Nikon Eclipse TE2000E filter block-equipped microscope (Nikon, Japan) coupled to a Carin Optoscan monochromator (Carin, UK) with a Prior Proscan *XYZ* motorized stage on an upright platform (Prior Scientific Ltd., UK). A cage incubator (H201 Okolab S.r.l., Italy) was attached to the microscope stage to maintain constant culture conditions (at room temperature 20 ± 2°C, and atmospheric concentrations of CO_2_ and O_2_). Cells from the ChlSP strain cultured in U-free BG-11 medium were placed in multiwell plates (Nunclon, Denmark) pretreated with poly-L-lysine (a substratum that limits microalgae movement in order to facilitate the subsequent analysis) 24 h before the beginning of the recording. Immediately before starting the recording, the culture medium was changed to Saelices medium; a control was maintained in U-free BG-11 medium. Cells were sequentially illuminated with monochromatic light from the monochromator at 365, 480, 547, and 635 nm. The light emitted was isolated by individual band pass filter blocks of 510, 520, 590, and 700 nm, respectively. Micrographs were taken using a high-resolution Hamamatsu CCD camera C4742-98 (Hamamatsu, Japan). The exposure times were <800 ms at each wavelength. All devices were controlled by MetaMorph software (Molecular Devices, USA). For each wavelength, stacks of three images taken 5 μm apart along the *Z-*axis were collected at 10 min intervals for 22 h. Time course data represent the maximal light intensity in a small elliptical region inside each algal cell. Cell vitality was evaluated by optic microscopy at the end of the experiment.

### U-uptake-capacity determination

The capacity for total U uptake was measured in the ChlSP strain and the selected ChlSG strain. Cells from both strains were grown in Saelices medium for 1 week, under the same environmental conditions mentioned above and grown for the same exposure time. Then, pellets of cells were harvested by centrifugation (at 2,000 g and 3 min). We removed the free U from the culture medium (i.e., all of the U that was not adsorbed on the cell wall or accumulated inside the cells) with five successive cycles of centrifugation and resuspension in fresh BG-11 medium. Finally, we obtained pellets of around more or less 7 × 10^7^ cells. To calculate the U distribution in the cells, differentiating the adsorbed and intracellular U faction, the pellets of ChlSG strain were separated according to two different procedures. Three pellets were used to evaluate total U in the cells, and the other three pellets suspended in 4 mL of BG-11 medium supplemented with 0.05% EDTA for 15 min to eliminate the loosely bound U fraction attached to the surface of the cells (Panak et al., 2000). In all cases, U concentration was measured in the acid-digested pellets by ICP-MS (VARIAN RedTop ICP mass spectrometer, Palo Alto, USA; García-Balboa et al., 2013).

U-uptake experiments were conducted in triplicate in cell culture flasks of 650 mL (Greiner, Bio-One Inc., Longwood, USA) with 85 mL of BG-11 enriched with 4 mg L^−1^ of U. Initially, cells were inoculated at a density 5 × 10^4^ cells mL^−1^ and allowed to grow under the aforementioned laboratory conditions up to late stationary phase (up to 48 days). The experiments were carried out for a long period of time and increasing cell densities to maximize the U depletion through adsorption and uptake by the microalgae cells. The same ChlSG culture, propagated in BG-11 without U for 4 months, was used as the parental culture for the cell inoculum of each replicate. U-uptake measurements were made at different times (initially at 2 h and afterwards at 3, 9, 12, 24, 36, and 48 days), throughout the culture growth cycle. Cell pellets were gently harvested by centrifugation (at 2,000 g and 3 min) from 5 mL microalgae culture. The resultant supernatant was filtered and acidified for direct quantification of total U. Harvested pellets were measured for the U content by wet acid digestion and drying to constant weight (acid mixture containing nitric acid 65% and hydrogen peroxide 30% (HNO_3_/ H_2_O_2_) (Suprapur, Sigma-Aldrich) at a 4:1 v/v ratio, and heated at 85°C during 12 h in Parr's acid digestion vessels (Moline, USA).

### Statistical analyses

A pairwise *t*-test was used to compare treatments (e.g., comparison of ancestral and selected strains and comparison of U distribution among the algae cells). Statistical differences in time-dependent U uptake by the cells and depletion from the supernatants were tested using one-way ANOVA and Tukey *post-hoc* analyses. Unless otherwise stated, results are expressed as the mean values of three replicates with standard deviation. Significance was considered at *P* < 0.05.

## Results

### Isolation and phylogenetic analyses of U-uptake candidate microalga strain

Samples from over 20 mining ponds were collected from diverse water bodies located in the Saelices mining area, Salamanca, Spain. These water samples contained U in concentrations ranging from 25 to 48 mg U L^−1^, and many other environmental stressors such as dissolved elements [in order of concentration Zn (17.5–87.24 mg L^−1^), Cu (0.85–16.44 mg L^−1^), As (0.15–1.69 mg L^−1^), and Pb (5.58–12.2 μg L^−1^)], pH ≈3 and radioactivity of 4 μSv h^−1^ at 1 m distance (García-Balboa et al., 2013; Baselga-Cervera, 2017). In particular, water samples refereed as Saelices presented as follows: 25 mg U L^−1^, 17.5 mg Zn L^−1^, 0.85 mg Cu L^−1^, 0.15 mg As L^−1^, and 5.58 μg Pb L^−1^. Further semi-quantitative analysis of element distribution, excluding U, was performed in Saelices water samples by means of a spectrophotometer, using ICP-MS, identifying: Mn, Mg > Ni, Ce, Nd, Sm, Eu > S, Sr, Y, Eu, Gd, Tb, Dy, Ho, Er, Yb, Tm, Lu, and Re. Concentrations ranged from 100 to 500 ng L^−1^.

**Figure 1:**
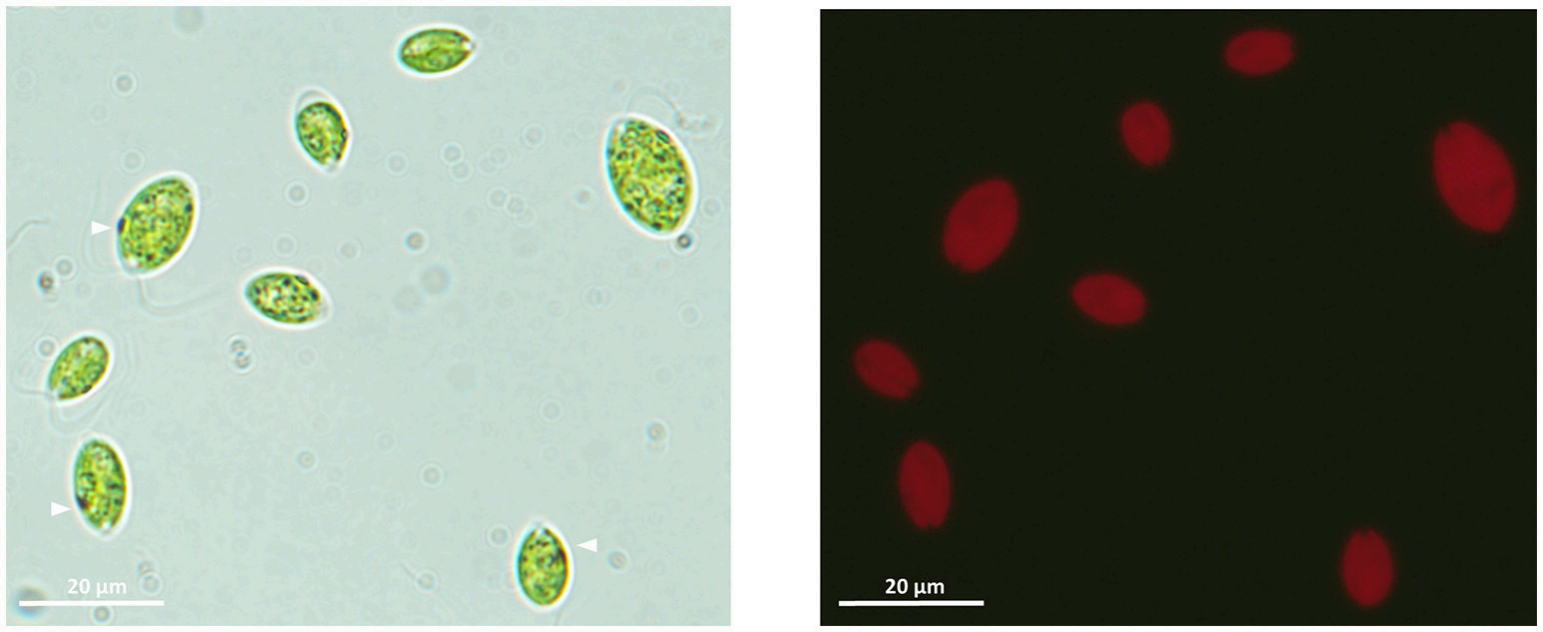
Light microscope (100×) images of the ChlSP microalgae isolated from Saelices mine tailings ponds. Left-hand image, vegetative single cells with the apical flagellar end (arrowheads point to the photoreceptor-organelle eyespot). Right-hand image, chlorophyll auto-fluorescence of vegetative ChlSP cells.

Isolation of single-species cultures was confirmed by optical microscopy, and morphological comparisons with previously described species, following the methodologies of AlgaeBase, suggested that the isolated microalgae species belonged to the genera *Chlamydomonas, Dictyosphaerium, Euglena*, and *Pleurosigma*. The species of the genera *Chlamydomonas* were present as by far the most dominant species in all the U mine water samples, whereas not many microalgae are capable of growing in U mine tailings water with high levels of U contamination as previously described in *Chlamydomonas reinhardtii* and *Dictyosphaerium chlorelloides* (García-Balboa et al., 2013). Moreover, the *Chlamydomonas* sp. isolate grew rapidly in U mine waters and was able to grow in U-enriched media under laboratory conditions, and it was the only species with a rapid motility and positive phototaxis. We designated the U-mining-tolerant strain of the genera *Chlamydomonas* hereafter as ChlSP (Figure 1); the strain was placed in the Spanish Algae Bank [Banco Español de Algas (BEA)], deposit D04-12 (Costas Costas et al., 2012; Baselga-Cervera, 2017). The isolate ChlSP among the four isolated candidates was revealed as the strain that met the required criteria for U uptake and artificial selection (i.e., a fast growth rate with U and in U mining tailings waters, no special nutritional requirements, feasible recombination under laboratory conditions, a large cell wall surface and motility)

**Figure 2:**
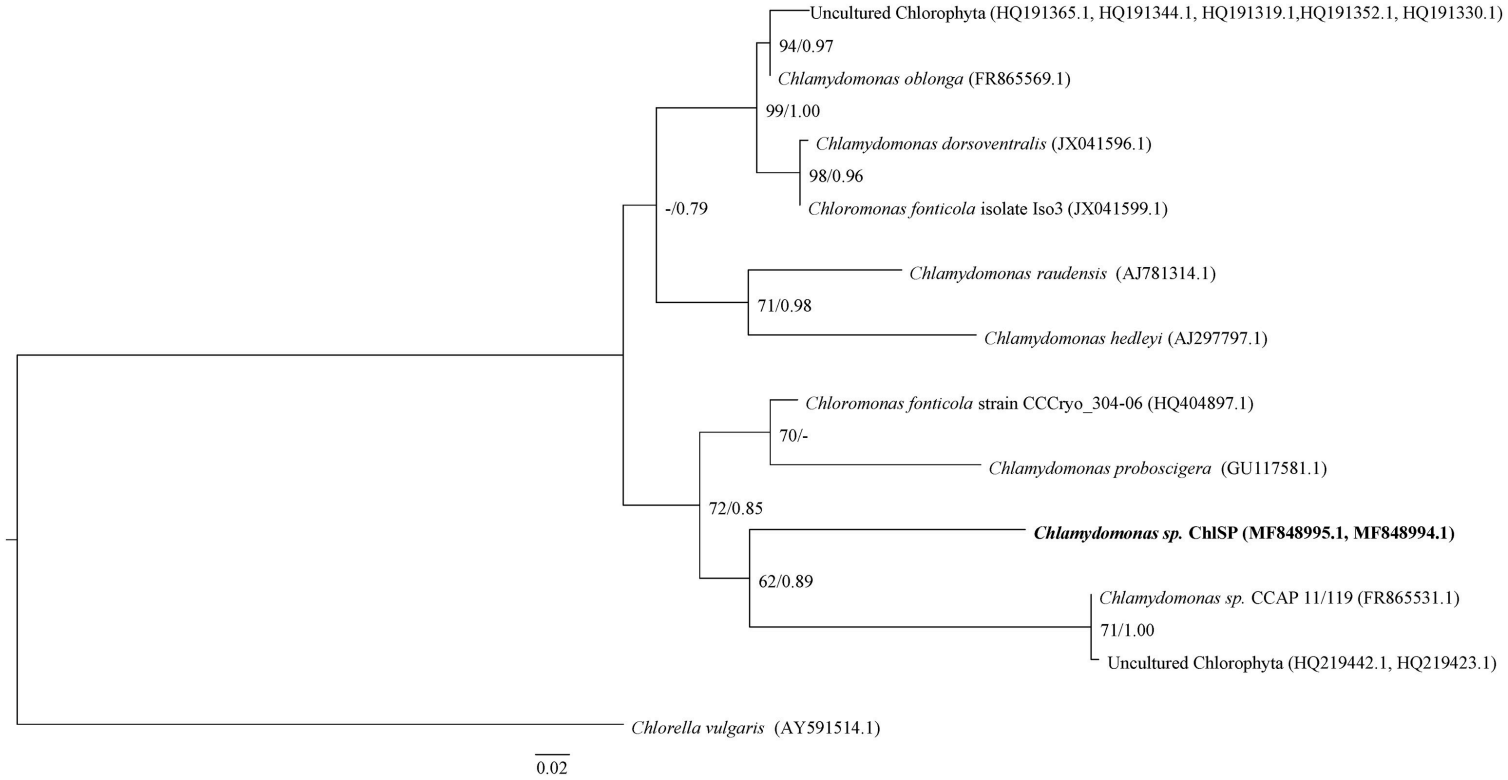
ML tree inferred based on the rDNA ITS and 5.8 S sequences. Numeric values at nodes are the ML BP (bootstrap percentage)/BI PP (posterior probability). Dashes (-) indicate < 50% percentage values. –Ln likelihood was 1423.138; the substitution model chosen was GTR+G; nucleotide frequencies A = 0.2388, C = 0.2759, G = 0.2330, T = 0.25211; substitution model rate matrix (R(a)[A-C] = 2.2194, R(b)[A-G] = 3.4983, R(c)[A-T] = 2.2692, R(d)[C-G] = 1.1608, R(f)[C-T] = 8.7438); gamma shape parameter = 0.310. Scale bar = 0.02 substitutions per site. Boldface indicates the new species, the ChlSP strain, described in this study.

To specify the identity of ChlSP, the ITS1-5.8S-ITS2 region was successfully sequenced from genomic DNA samples extracted from a pellet of algae. The ITS1 and ITS2 sequences have proven to be the best candidates for use as indicators of phylogenetic relationships among green algae and, in particular, *Chlamydomonadaceae* (Gonzalez et al., 2001; Pocock et al., 2004; Possmayer et al., 2016). The sequences amplified were 709 bp (including 273 bp of 18S rRNA and ITS1, 154 bp 5.8S rRNA gene, 268 bp ITS2, and 14 bp 28S rRNA gene) and 755 bp (including 295 bp of 18S rRNA gene and ITS1, 154 bp 5.8S rRNA gene, 268 bp ITS2, and 38 bp 28S rRNA gene), GenBank accession numbers MF848994.1 and MF848995.1, respectively. BLAST analyses (Zhang et al., 2000) against the nucleotide datasets in the NCBI database (http://www.ncbi.nlm.nih.gov/BLAST) placed the strain ChlSP firmly into the *Chlamydomonadaceae, Chlorophyta*, but produced matches below the expected (*E*) values and low identities (<92%). Sequences from different *Chlamydomonas* species, two *Chloromonas fonticola* and several uncultured *Chlorophyta* were highly similar to the two ChlSP sequences (Figure 2). Additionally, sequences from other algae genera, *Nephroselmis, Tetracystis*, and *Haematococcus*, were found to distantly match with the ChlSP ITS2 sequences. The alignment for ITS1-5.8S-ITS2 regions for ChlSP sequences, unique to this study, and those most closely related matches obtained from the GenBank database were selected for the phylogenetic analysis, and *C. vulgaris* (AY591514.1) was used as an outgroup. The ML tree was depicted with support, ML and BI phylogenetic inference methods were concordant and presented well-supported nodes (Figure 2). The monophyly of the strains studied was moderate to fully supported; although, relationships within and among strains/species were poorly resolved, placing the same species, *C. fonticola*, in different clades. ChlSP formed a district subline within a subclade with several unidentified *Chlorophyta* environmental sequences and an undescribed *Chlamydomonas* sp. from a culture collection. Astonishingly, ChlSP was sister to two cryophilic species isolated from Antarctica (*C. fonticola*) and the Artic (*Chlamydomonas proboscigera*). Interestingly, the other main subclade included symbionts found in foraminifers such as *Chlamydomonas hedleyi* (Pawlowski et al., 2001), environmental uncultured *Chlorophyta*, psychrophilic species (*C. fonticola* and *Chlamydomonas raudensis*) and species isolated from wastewaters (*Chlamydomonas dorsoventralis* and *Chlamydomonas oblonga)*.

### Morphological characterization

**Figure 3:**
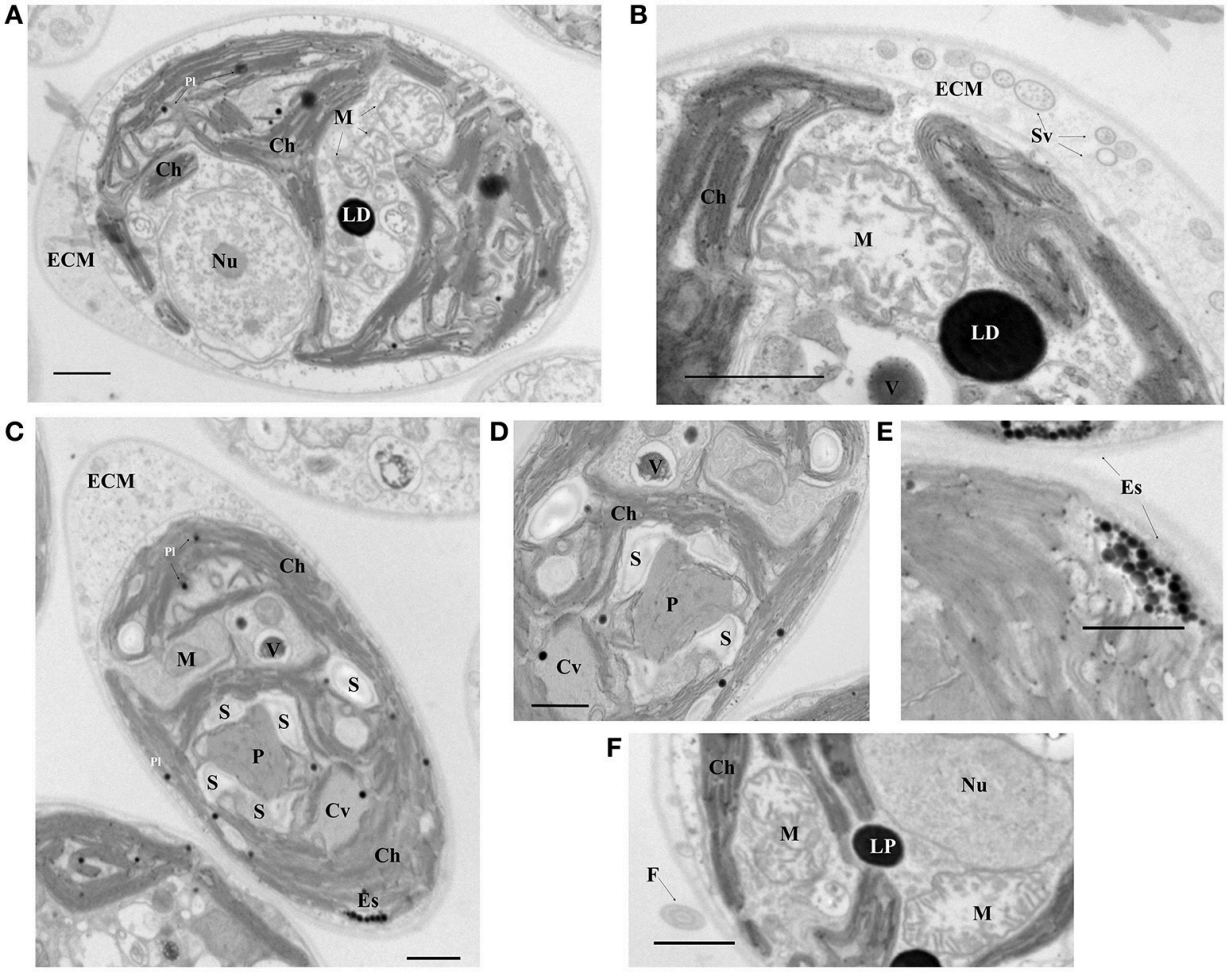
Electron micrographs of the ChlSP strain. **(A,C)** Transverse sections of a single cell; **(B,D–F)** close up of cell components—**(B)** cell wall and detail of a mitochondria, **(D)** pyrenoid, **(E)** eyespot, and **(F)** flagellum. Scale bars, 1.0 μm. (Ch, chloroplast; M, mitochondrial profiles; Nu, nucleolus within the nucleus; Pl, plastoglobules; LP, lipid droplets; P, pyrenoid; S, starch F, flagellum; Es, Eye spot; ECM, extracellular matrix; Sv, secretory vesicles; Cv, contract vacuole profile; V, vacuole with possible U precipitates).

The isolated strain ChlSP exists as biflagellate oval-shaped single cells, ranging from 8 to 12 μm in diameter, and the two equal flagella are ~1.5 times longer than the body diameter in length (Figure 1). Asexual reproduction results in the formation of a sporangium of 8–16 zoospores; sexual reproduction was recognized by direct observation of gametes, active mating and zygotes. Figure 3 illustrates the overall and cross-section transmission electron micrographs of ChlSP at 12,000 × to 40,000 × magnifications. The images show abundant H-shaped chloroplasts, occupying approximately half of the volume of the cell, with space stroma and large plastoglobules. The nucleus is located centrally or anteriorly between the chloroplast, in which the nucleolus can be differentiated. Several oversized mitochondrial profiles are easily recognizable in the cytoplasm (Figure 3). Cross-sections of the flagella reflect a tubule of 9+2 construction (Figure 3F), a common feature of this genera. ChlSP cells present a regularly disposed plasma membrane, surrounded by an extracellular matrix and bound by a wall sheath that contains embedded in the matrix round-shaped structures compatible with secretory vesicles. Starch was gathered as plates surrounding the pyrenoid, but not restricted (Figures 3C,D). Eyespots were small and irregular, located within the chloroplast in the flagellar proximal position (Figure 3E). Several vacuoles with electron-dense granular deposits could be identified in the cytoplasm (Figures 3B–D); these type of deposits are compatible with U phosphorus deposits, as previous studies of the ChlSP transmission electron micrographs have confirmed by EDX analysis (Baselga-Cervera et al., 2013; García-Balboa et al., 2013).

### Artificial selection for U uptake and location of U in the cells

In addition, we noted that it was very easy to induce sexual reproduction in ChlSP by nitrogen depletion. Shortly after nitrogen depletion, many active-mating gametes and numerous zygotes were observed in the culture. The strain ChlSP, after recombination, was transferred to Saelices medium. After exponential growth of this population with genotypic variability maximized by recombination, we selected cells by settling selection, considering that cells containing a higher U content should sink more quickly than those that less U accumulated. With this selection, we intended to increase the U-uptake capacity of the strain independently of the route of uptake (previous studies with ChlSP noted the capacity of both biosorption in the cell walls and bioaccumulation inside the cytoplasm of the cell; García-Balboa et al., 2013). When the cells were placed at the top of a burette, most of the cells remained near the surface, swimming toward the light. Slowly, cells started to sink, some of them reached the lower part of the burette in ~15 min. After several selection cycles, we used the fastest-sinking cells to find the ChlSG strain, which presented an exponential growth rate by asexual division of around 0.7 doublings day^−1^ in Saelices medium. These cells were always in the bottom of the cell culture flasks showing a phototactic response and presented a decreased ability to swim toward the surface, but their swimming was not impaired.

To estimate the selection response, we compared the efficiency of U uptake between the ChlSP and the ChlSG strains. ICP-MS analysis demonstrated that *Chlamydomonas* cells from the ChlSP strain took up 31.56 ± 1.87 × 10^−5^ ng U per cell [~4.30 mg U g^−1^ of dry biomass (DB)], whereas the cells from the artificially selected strain increased by ~1.5 times its ability to uptake U (46.49 ± 2.82 × 10^−5^ ng U per cell, ~6.34 mg U g^−1^ DB). Consequently, the response to selection (*R*) was extremely high (14.93 × 10^−5^ ng U per cell), corresponding approximately to 80% of the theoretical maximum response (*R*_max_ ≈58 × 10^−6^ ng U per cell, ≈7.9 mg U g^−1^ DB). Moreover, heritability was also very high (*h*^2^ > 0.8), indicating that most of the variability in the U uptake could be due to genetic causes, whereas the other causes involved (e.g., environmental forces) have scant influence. Moreover, the ChlSG pellets treated with 0.05% EDTA resulted in 3.39 ± 0.2 × 10^−5^ ng U per cell, ~0.46 mg U g^−1^ DB. Compared to the untreated pellet, 6.34 mg U g^−1^ DB, around 90% of the U uptake by the cells of the ChlSG strain was released by the EDTA extraction. Therefore, only a small part, the remaining ≈10%, was irreversibly bound to the ChlSG cells by bioaccumulation.

### *In vivo* visualization of the U-uptake process

**Figure 4:**
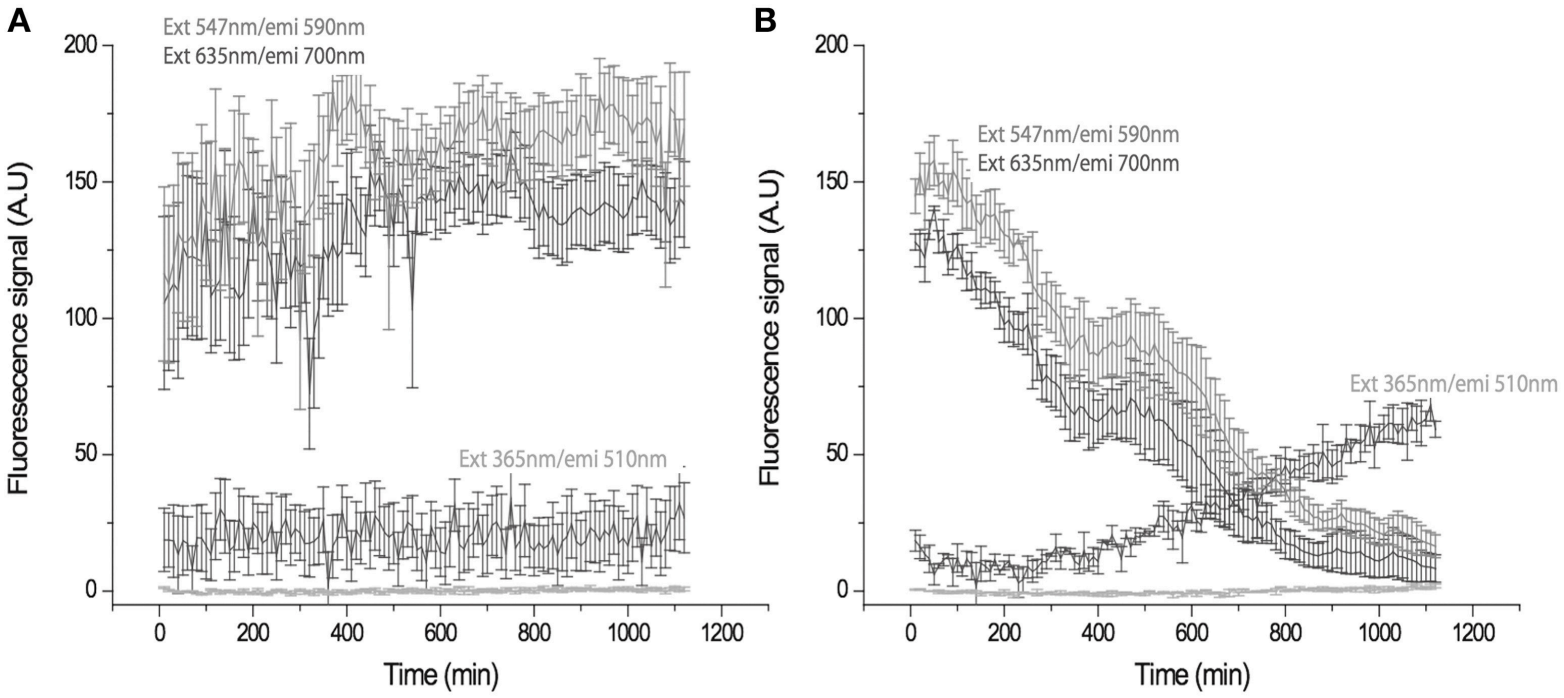
Time-trace showing fluorescence signal in arbitrary units (A.U.) of the cells at different emission longitudes in the absence **(A)** and presence of U **(B)**. **(A)** The control emission remains constant, cells emitted at 590 and 700 nm, after 20 h. **(B)** The U-exposed sample shows a gradual change in the emission of the cells from 590 nm and 700 nm to 510 nm. Changes are compatible with U uptake being appreciable at 10 h. Maximum intensity is reached at 20 h. (ext, excitation; emi, emission).

U presents natural fluorescence with a UV excitation wavelength in the range of 260–380 nm (Hashem et al., 2013), maximal fluorescence values with wavelengths ranging from 494 to 565 nm, and a detection limit of 0.1 μg U L^−1^ under UV light exposure (Moulin et al., 1997). U has been detected in several different organisms by fluorimetric techniques (Premadas and Srivastava, 1999). Therefore, we visualized the *in vivo* ChlSP U uptake in real time by exposing cells to UV light and monitoring the fluorescence emission of the cells. Cells were monitored by chlorophyll emitted fluorescence. Although U and chlorophyll have emission spectra that are very close, U has a characteristic maximum of emission at 509 nm, far from the maximum emission of chlorophyll *b* at 670 nm. After 20 h of recording, the movies generated [control (Video S1) and study (Video S2) videos] were analyzed using Metamorphic software. The time-lapse image analysis revealed a significant increase in the fluorescence signal at 510 nm in the cells in the presence of U, when cells were exposed at 365 nm for 10 h; after 20 h, the fluorescence signal approached the maximum level (Figure 4B). An emission wavelength at 590 and 700 nm, chlorophyll emission ratio, presented a decreasing slope over time in the case of the study sample (Figure 4B). However, in the absence of U, no significant variation in the fluorescence signals was observed (Figure 4A), as was reflected by the two more representative fluorescence signals from the chlorophylls *a* and *b*.

### U uptake by ChlSG

**Figure 5:**
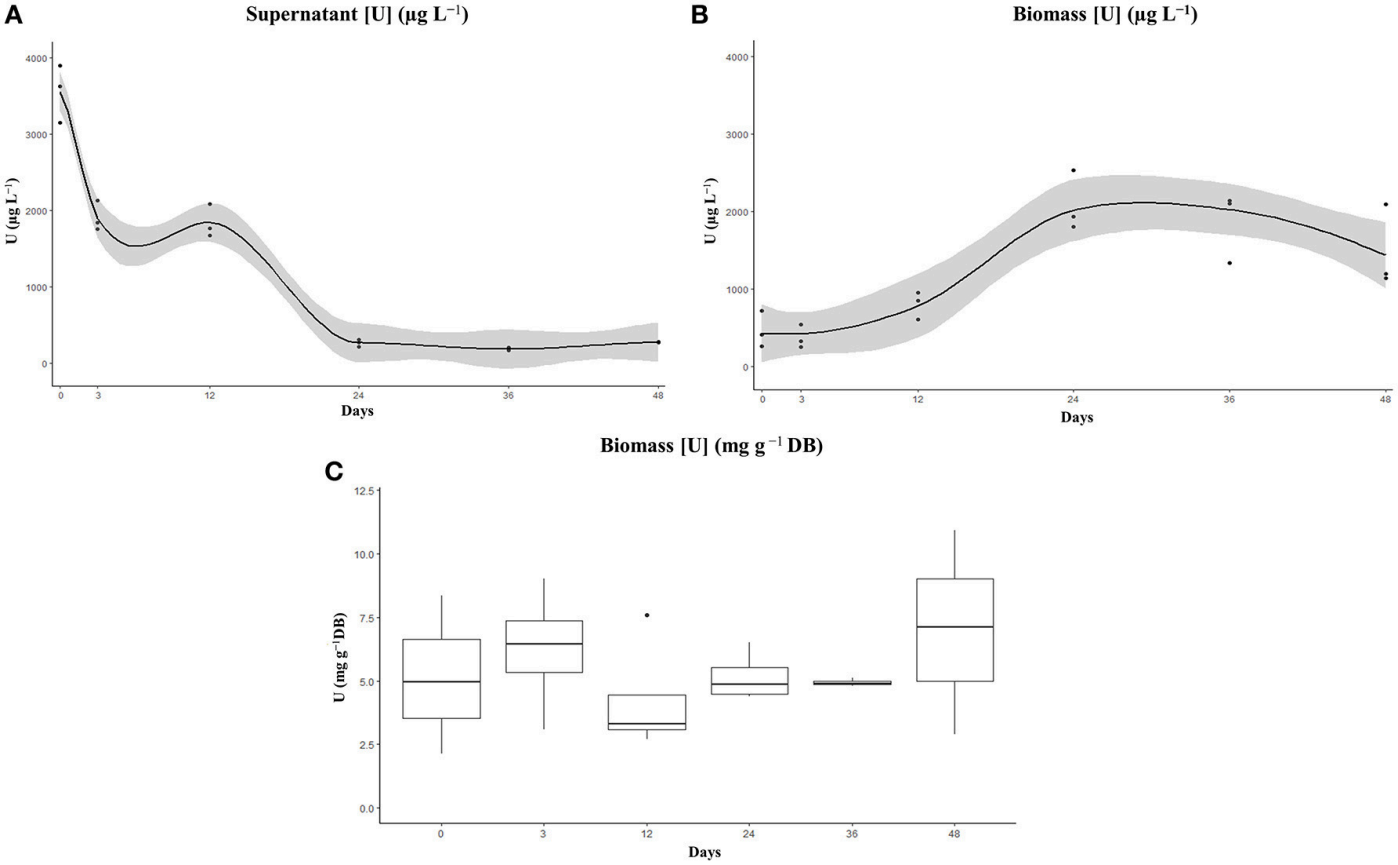
Elimination (recovery) **(A)** and cell uptake **(B)** of U from BG-11 medium supplemented with 4 mg U L^−1^ by the artificially selected ChlSG strain after 48 days in free suspension. The amount of U significantly decreased in the supernatant **(A)** and the biomass **(B)** showed a positive slope in the U uptake over time. No significant differences can be observed in terms of U uptake g^−1^ DB over time **(C)**, with a mean value of ~6 mg U g^−1^ DB of ChlSG.

During the incubation of ChlSG, the U dissolved in water was reduced significantly in BG-11 medium containing 4 mg L^−1^ of U at pH 5. In this study, we did not distinguish between adsorption and bioaccumulation, but rather analyzed the total metal concentration within the biomass and the liquid phase of the dilution over time, as we were *a priori* concerned with the total U remediation potential of the ChlSG strain. The pH of the cultures at the end of the experiment did not exceed 7 and no precipitation was observed. Most of the U was removed from the water by direct uptake by means of growing U-resistant cells (ChlSG strain). U uptake was plotted as a function of time (Figure 5), which was measured as the total U concentration (μg L^−1^) in the medium (supernatant) and in the microalgae biomass. A very good agreement was obtained in the supernatant (Figure 5A), the amount of U from the initial 3–4 mg L^−1^ at 1 h of exposition changed to > 0.3 mg L^−1^ at the end of the experiment (determination coefficient *r* = −0.85 and *P* < 0.001). The biomass showed a positive slope in the U uptake over time (*r* = 0.6 and *P* < 0.01; Figure 5B).

Of note, there is a correlation between the population growth cycle and the U removal in terms of total U concentrations in the liquid phase and in the cell biomass. Regarding the liquid phase, a curve with a constant decreasing slope can be observed during the exponential growth phase, the first 24 days of the experiment, in the total U concentration. From day 24 up to day 48, screened at the stationary growth phase, no significant U removal was observed for the liquid phase. Significant differences in the supernatant can be observed between measurements in the supernatant (one-way ANOVA *F*-value = 170.1, *P* < 0.001). There are statistical differences between time 0 and the rest of the measurements, as well as between days 3 and 12 and the measurements later on. However, there were no significant differences between days 3 and 12 (*P* = 0.796), and between the three last measurements (*P* = 0.99; Figure S1A). There was also a statistically significant difference between U-binding biomass measurements as determined by one-way ANOVA (*F*-value = 12.4, *P* < 0.001). The biomass measurements after a short lag phase, the first 72 h, presented a constantly increasing slope related to the log phase up to day 24, the Tukey *post-hoc* test did not show statistical significance of measurements at 0, 3, and 12 days, but revealed significant differences to day 24 (*P* < 0.001; Figure S1B). After day 24, no significant differences were observed (*P* > 0.1), the U concentration in the biomass remained constant up to the late stationary phase in which a decline in the sequestration capacity could be observed, probably related with cell death. In terms of U uptake per unit of cell biomass, measurements ranged from 2.5 to 14 mg U g^−1^ DB over time (no significant differences, *F*-value = 0.92, *P* > 0.5), with a mean of ~6.3 mg U g^−1^ DB of ChlSG (Figure 5C). If cell growth is sufficiently high, more biomass will be available for U uptake and will have a positive impact on overall bioremediation.

## Discussion

### Saelices U mine tailings ponds: a source of species novelty

Anthropogenic extreme environments represent unique reservoirs for studying rapid evolution, understanding the intricate mechanisms underlying survival of extremophiles and biotechnological potential (van den Burg, 2003; Anitori, 2012; Bell, 2012; Raddadi et al., 2015; Rampelotto, 2016). U operations in the Saelices mining area have rendered metal-rich waters, generally acidic and with radionuclides (Lozano et al., 2000; Antunes et al., 2007; Neves and Matias, 2008; Baselga-Cervera et al., 2013; García-Balboa et al., 2013), which have arisen from mining activities and residues. Our wider screening of metals in water tailings showed rare-earth elements (like erbium and europium), as previously described in U tailings in Sillamäe, Estonia (Kosynkin and Nikonov, 2000). All these peculiarities have rendered the aquatic environment of the Saelices mine an anthropogenic extreme environment.

These types of ecosystems are in fact almost barren, only a few microalgae species have been identified and no macroscopic life was observed. Unlike natural extreme environments, which are relatively stable for a long period, anthropogenic extremes arise in short time-scales; mining activities in Saelices were started in July 1974 by *Empresa Nacional del Uranio* (ENUSA) (Spanish National Uranium Company). Flora and fauna are pre-adapted for the extreme conditions and the pressures imposed for subsistence enable just a narrow number of ways for this. In the case of prokaryotes, previous studies have identified the presence of several bacterial species (de Silóniz et al., 1991), but cyanobacteria were not identified, presumably due to the highly acidic profile of the water (Steinberg et al., 1998). Among the different microalgae isolates, we delved into the study of the strain ChlSP. One of the most striking physiological characteristics of ChlSP is that it is able to thrive in U-rich mine acid waters and bioaccumulate U. This *Chlorophyta* strain shares the general *Chlamydomonas*-like morphology, physiology and ultrastructure; a similar size and shape of oval apical bi-flagellar cells with eyespot apparatus; a basal pyrenoid embedded in a mass of chloroplasts; and presents motility, phototaxis, and sexual reproduction. Similarly, the phylogenetic study based on the nuclear ITS-5.8S rDNA region sequences showed divergence from the other close species of the genus *Chlamydomonadae*, indicative of an isolated phylogenetic position and, thus, a split from the nearest neighbors. The ChlSP strain was placed on a solitary branch in a clade with two unidentified *Chlorophyta*, nested within a clade as sister group to *C. fonticola* and *C. proboscigera*. Affiliations to the species were not statistically supported in any of the phylogenetic analyses, the ChlSP strain might represent a new species within the genus. Henceforth, we propose to call our extremotolerant strain *Chlamydomonas saelices* sp. nov. A BLAST search showed close relations with several *Chlamydomonas* and several uncultured *Chlorophyta* isolates, including previously related foraminifer symbiont species and psychrophilic extremophiles (Pocock et al., 2004). These relationships raise interesting questions regarding the evolutionary history of the ChlSP strain and convergence adaptation in extremophiles.

In green algae genera, *Chlamydomonas* is one of the largest, encompassing more than 800 species. The genus diversity comprises species found in stagnant water, freshwater, marine water, soils, snow, and even foraminiferal symbionts (Brodie and Lewis, 2007). However, precise determination at the species level is often difficult and, in many cases, the species diagnosis remains incomplete as outlined in the BLAST sequences search. *C. saelices* sp. nov. might represent an example of a rarely accessible species, an undetermined isolate or a newly evolved strain. Alternatively, new genera and species of green microalgae are discovered every year and the diversity within these microorganisms remains largely unexplored. Only a fraction of the global microbial diversity has been explored, due to the limited sampling of Earth, or been described (May, 2011; Mora et al., 2011; Locey and Lennon, 2016).

Enticed by the extremes, scientists have been intrigued by the “life on the edges.” Since the discovery of extremophiles, they have been targets for the study of life's basic processes and life performance under selective conditions. Regarding the peculiarities of the U-mine acid drainage environment, the ChlSP strain can be considered an example of a strain from a human-polluted extreme environment resulting in particular adaptations with taxonomic and functional novelties. This strain is pre-adapted to the stressors present in U mine waters and might present several adaptive features, apart from the U bioremediation application explored in this work, correlated with bioremediation and biotechnological U industrial needs. Highly polluted environments represent a major ecological hazard, but also a hotbed source of novel extremotolerants and interesting sites for microorganism bioprospection.

### Artificial selection of microbial eukaryotes

Artificial selection and domestication of microorganisms, although in many cases unintentional, has long been carried out in relation to food, beverages, and biofuel production (Libkind et al., 2011). However, microbial domestication and selection only gained interest in the last 25 years, and an ever-increasing number of adaptive laboratory evolution experiments (Mitchell et al., 2009; Dragosits and Mattanovich, 2013; Jeong et al., 2016) and selection studies have taken place (Kuthan et al., 2003; Buckling et al., 2009; Liti et al., 2009; McLoon et al., 2011; Eydallin et al., 2014). Several works outline the potential of these approaches in biotechnological engineering and cost-efficient production of microalgae (Bonente et al., 2011; Duong et al., 2012; Cazzaniga et al., 2014; Cheng et al., 2014).

A major limitation of biotechnological procedures is that many microorganisms are not pre-adapted to the new environment. Consequently, the first step toward selecting live microbes in biotechnology processes is obtaining strains tolerant or domesticated to the imposed conditions. Several examples of selection procedures, i.e., a modification of the classical fluctuation analysis (Luria and Delbrück, 1943), allowed rapid selection of microalgae mutants resistant to a wide range of conditions (Baos et al., 2002; Garcia-Villada et al., 2002; López-Rodas et al., 2006b; Costas et al., 2008, 2013, 2014; Marvá et al., 2014; Rouco et al., 2014; Baselga-Cervera et al., 2016). Recent data suggest that a mutation in a single gene can produce resistance to high doses of U (García-Balboa et al., 2013). However, tolerance for mine waters is probably a complex quantitative trait, which is the result of the expression of numerous genes, each with a small effect, modified through the interaction between a particular genotype and the external and internal environment. Such quantitative traits are those that can be enhanced by artificial selection rather than by genetic engineering.

In the artificial selection of U-uptake features performed in this work, we have taken advantage of the work already performed by nature, which has led to the isolation and selection of metal-rich-resistant genotypes during the last 40 years. The isolated ChlSP strain, unlike other coddled domesticated microbial strains for industrial biotechnology, is endowed with features for competitive natural ecosystems and fits for *in situ* bioremediation. Then, by selection in the isolation medium (mine water from Saelices), making use of the available diversity of ChlSP after recombination, we selected genotypes with mutations or up-regulated genes to attain higher U concentration uptake, either passively or actively, by using an indirect method; such genotypes were selected by increased sinking speed. We presumed that by selecting those cells that sink faster in Saelices medium, we were selecting for cells with high density and/or size and, thus, presenting more U bound and/or intracellular U. Finally, we obtained the selected strain ChlSG by growing asexually (without recombination) the genotypes that sank faster and presumably had a higher ability to take up U. Results showed that the artificial selection of *Chlamydomonas* sp. opens up promising new prospects for U bioremediation. The artificially selected strain exhibited nearly 1.5 times the ability to sequestrate U compared to the ancestral wild-type ChlSP population in mine-water conditions. Furthermore, the response to selection obtained after the artificial selection was close to 80% of the theoretical maximum response. The downside of the selection system is that despite the positive response, we cannot establish the traits or genes that lie beneath the feature of increased U sequestration. Moreover, only 1.5-fold increase does not present a high potential for bioremediation.

Perhaps the fact that has hindered the development of artificial selection in microalgae is that they are mostly haploid with asexual reproduction. Artificial selection requires first obtaining genetic variability, by mutation or interbreeding, from which desired genotypes are selected. This is often a laborious process; frequently the endogenous genes or sexual cycles are lacking. In an attempt to bypass such a lengthy selection method, genetic engineering has been used with microalgae. Although advances in the genetic engineering of several microalgae species have been achieved during the last decade (Zaslavskaia et al., 2001; Fuhrmann et al., 2004; Kumar et al., 2004; Poulsen et al., 2006; Sun et al., 2006; Molnar et al., 2009; Radakovits et al., 2010), an increased number of authors have suggested that the full potential of microalgae artificial selection can be fully implemented only if conventional selection methods become firmly established (Chepurnov et al., 2004, 2008). There is ample evidence that even haploid microalgae are ideal for enhancement by artificial selection. First, natural populations of microalgae contain ample genetic variability for useful quantitative traits (Brand, 1981, 1989; Costas, 1990; Carrillo et al., 2003; Bañares-España et al., 2006, 2007). Second, phytoplankton organisms show very high heritability, i.e., genetic contribution to the phenotypic variability of quantitative traits (López-Rodas et al., 2006a; Rico et al., 2006; Bañares-España et al., 2007, 2013). Third, it has already been demonstrated that microalgae respond rapidly to artificial selection (Huertas et al., 2010, 2011; Romero-Lopez et al., 2012; Rouco et al., 2014).

The cells from the enhanced ChlSG strain were able to rapidly proliferate in water samples from the Saelices U mine. This fact is a definite advantage for conducting future bioremediation studies in the laboratory and *in situ*, with this artificially selected strain. Furthermore, the absence of genetic manipulation and the selection of native microalgae make the selected organisms of particular relevance for environmental remediation purposes in the Saelices mine, avoiding regulatory scrutiny due to legal restrictions on genetically modified organisms and the introduction of invasive species (Glaser and Glick, 2012; Snow and Smith, 2012; Gressel et al., 2013). Consequently, the artificial selection of native species seems to be an interesting procedure for bioremediation in extremely contaminated environments.

### U sequestration: potential applications for bioremediation

The enhanced ChlSG strain is able to sequestrate up to 6.3 mg U g^−1^ DB from Saelices medium, during exponential growth. Moreover, the ChlSG strain was able to remove dissolved U from 4 mg L^−1^ solution down to 5% within 24 days in a non-continuous culture (Figure 5). From day 24 to day 48, during the stationary phase, no significant amounts of U were released from the cells to the media. Regarding the biomass, the maximum efficiency of removal was achieved at 24 days and day 48 values suggest an incipient desorption, probably related to the beginning of cellular death at late stationary phase. The uptake in terms of U g^−1^ DB ranged around 6 mg, and no significant differences were found along the growth curve in batch culture (Figure 5). However, the U bioavailability is affected by the solution, influencing the U speciation. The pH increase, up to pH ≈7 in the uptake experiment, changes the speciation from predominantly uranyl ion or hydroxyl complexes to soluble carbonate complexes (Choppin et al., 2002; Newsome et al., 2014) that might affect U mobility and adsorption. These results suggest that the efficiency of the cells, in relation to U recovered, did not change over the culture growth curve, and there is no evidence of competition with the different metals identified in Saelices medium. Nevertheless, in our model, we have not specifically investigated the possible dynamics of competition between other dissolved metals. Over the course of the experiments with Saelices water, we did not observe precipitates in the water phase, despite the pH increase (pH < 5). Possible co-precipitation, related with the pH increase mediated by microalgae, is one of the potential drawbacks of microalgae in mine-water management. Mine waters are complex mixtures of dissolved metals, organic, and inorganic compounds; the intricate interactions of these chemical solutions require further study to understand the biochemical interactions.

As revealed by the analytical approach, two possible mechanisms for U uptake could take place in the ChlSG strain: biosorption on the cell wall and bioaccumulation within the cytoplasm. The higher percentage of total U bound to the ChlSG cells (above 90%) was released by EDTA extraction, only a very small quantity, ~0.46 mg U g^−1^ DB, was irreversibly sequestrated. These results are in agreement with previous ones obtained using electron microscopy dispersive X-ray spectroscopy (García-Balboa et al., 2013; Baselga-Cervera, 2017). Both mechanisms could be hypothesized as part of a process performed by the algae as a defense against the toxicity of U. Heavy metals like U exert their toxic effect by competing with essential metals for active enzymes or membrane protein sites and by reacting with biologically active groups (Vasconcelos and Leal, 2001). Members of the genus *Chlamydomonas* have a cell wall that consists mainly of negatively charged polysaccharides and carbohydrates that attract cations such as Zn, Al, or U (Kalin et al., 2005), via a non-selective process. The cell wall represents a barrier that blocks the penetration of U, given that U has no biological function. However, if any amount of U is able to penetrate inside the cell, *Chlamydomonas* sp. is able to develop mechanisms of detoxification that sequester U in non-toxic forms, e.g., U internalization in association with granules formed by metal-binding proteins (Simon et al., 2011). Evidence of U internalization does not occur in all microalgae species, like in diatoms (Goldberg et al., 1998) or *Chlorella* (Horikoshi et al., 1979), and can be lethal to the cells, such as in *Pseudomonas fluorescens* (Krueger et al., 1993). The underlying process and the advantage of active intracellular U uptake by the cells is still unclear.

From an environmental engineering point of view, both the biosorption and intracellular accumulation noted in the ChlSP strain, present strengthens and weaknesses. In natural waters, uranyl ion [U(VI)-${\text{UO}}_{2}^{2+}$] is the predominant form of U in surface waters (Osmond and Ivanovich, 1992; Markich, 2002) and the most toxic form (Fortin et al., 2004), which is highly dependable on hardness and pH. U binding in the cells wall is a non-selective process and would occur even with dead microalgal biomass, but the U bound is directly influenced by the pH, the water hardness and the distribution of free uranyl ions (Markich, 2002; Fortin et al., 2007; Goulet et al., 2015). Previous works have related lower pH and an increase of water hardness with a decrease on the toxicity of dissolved U (Franklin et al., 2000; Charles et al., 2002), probably related with a reduction of uranyl ion bioavailability to bind to the surface of the cell (Riethmuller et al., 2001). Intracellular bioaccumulation requires an energy source and living cells, but the once internalized, the U is precipitated or concentrated in vacuoles.

Several biological methods, both passive and self-maintaining systems, have been used to treat U-rich waters and leachates, such as ion exchange, sorption on biological surfaces, or bioaccumulation by intracellular sequestration. The systems proposed include biowaste, bacteria, fungi, and algae capable of binding and uptake of U from the water media. The efficiency of U uptake described in the literature varies greatly among different species of microorganisms (Table S1 compiles the studies of U uptake mediated by microorganisms). The high proportion of negatively charged ionic groups in the cell wall makes microalgae more favorable to bio-adsorption of U cations than other organisms, active functional biopolymers or other chemical compounds (Yusan and Akyil, 2008; Tripathi et al., 2013). The limits for microalgae and cyanobacteria species tested ranged from 2.3 mg U g^−1^ DB in the green alga *Chlorella* to 77 mg U g^−1^ DB in the cyanobacterium *Anabaena* (Vogel et al., 2010; Acharya et al., 2012), detailed data from the literature are shown in Table S1. The selected ChlSG strain showed a performance of 6.3 mg U g^−1^ DB for U uptake.

Considering the particularities of our enhanced strain ChlSG, although this strain is not the most efficient based on previous studies (Table S1), it might be ideal for U removal from the waste stream and tailings ponds in Saelices mine. The strengths of the proposed system compared with those previously described, are: (i) living cells provide new organic material for sequestration, depending on the degree of cell growth, especially suitable for continuous removal; (ii) it presents active and passive U uptake by sorption in the cell walls and intracellular accumulation; (iii) the cells thrive in metal-rich acid mine waters; (iv) the cells are artificially selected native microorganism; and (v) it has side advantages of microalgae production, such as carbon dioxide sequestration, oxygen production, secondary resources like U from bioleaching, etc. Moreover, for future directions in the study of U anthropogenic extreme environments, other extremotolerant microbes can be investigated, the possibility of U recycling and secondary use from the U tailings residues can be assessed, and further research can be carried out to investigate the implications of the two routes of U uptake, bioaccumulation and biosorption.

## Author contributions

BB-C, CG-B, EC, and VL-R: Conceived and designed the experiments; BB-C, CG-B, and JR-L: Performed the experiments; BB-C, CG-B, EC, and VL-R: Analyzed the data; JR-L, CG-B, and BB-C: Contributed reagents, materials, analysis tools; VL-R, BB-C, JR-L, CG-B, and EC: Wrote the paper or made an important intellectual contribution.

### Conflict of interest statement

The authors declare that the research was conducted in the absence of any commercial or financial relationships that could be construed as a potential conflict of interest.

## Abbreviations

BI: Bayesian inference
DB: dry biomass
ICP-MS: inductively coupled plasma-mass spectrometry
ITS: internal transcribed spacer
ML: maximum likelihood.
