# Supplementary material for: Improvement of the Uranium Sequestration Ability of a Chlamydomonas sp. (ChlSP Strain) Isolated From Extreme Uranium Mine Tailings Through Selection for Potential Bioremediation Application
Source: Front Microbiol. 2018 Mar 21;9:523. doi: 10.3389/fmicb.2018.00523 (PMC5890155; doi:10.3389/fmicb.2018.00523)
Supplement: Supplementary file 1 [file DataSheet1.DOCX]

Supplementary Material

Improvement of the Uranium Sequestration Ability of a *Chlamydomonas* sp. (ChlSP Strain) Isolated from Extreme Uranium Mine Tailings through Selection for Potential Bioremediation Application

**Beatriz Baselga-Cervera, Julia Romero López, Camino García-Balboa, Eduardo Costas & Victoria López-Rodas***

*** Correspondence:** Corresponding Author: vlrodas@ucm.es

# Supplementary Figures

## Supplementary Figures


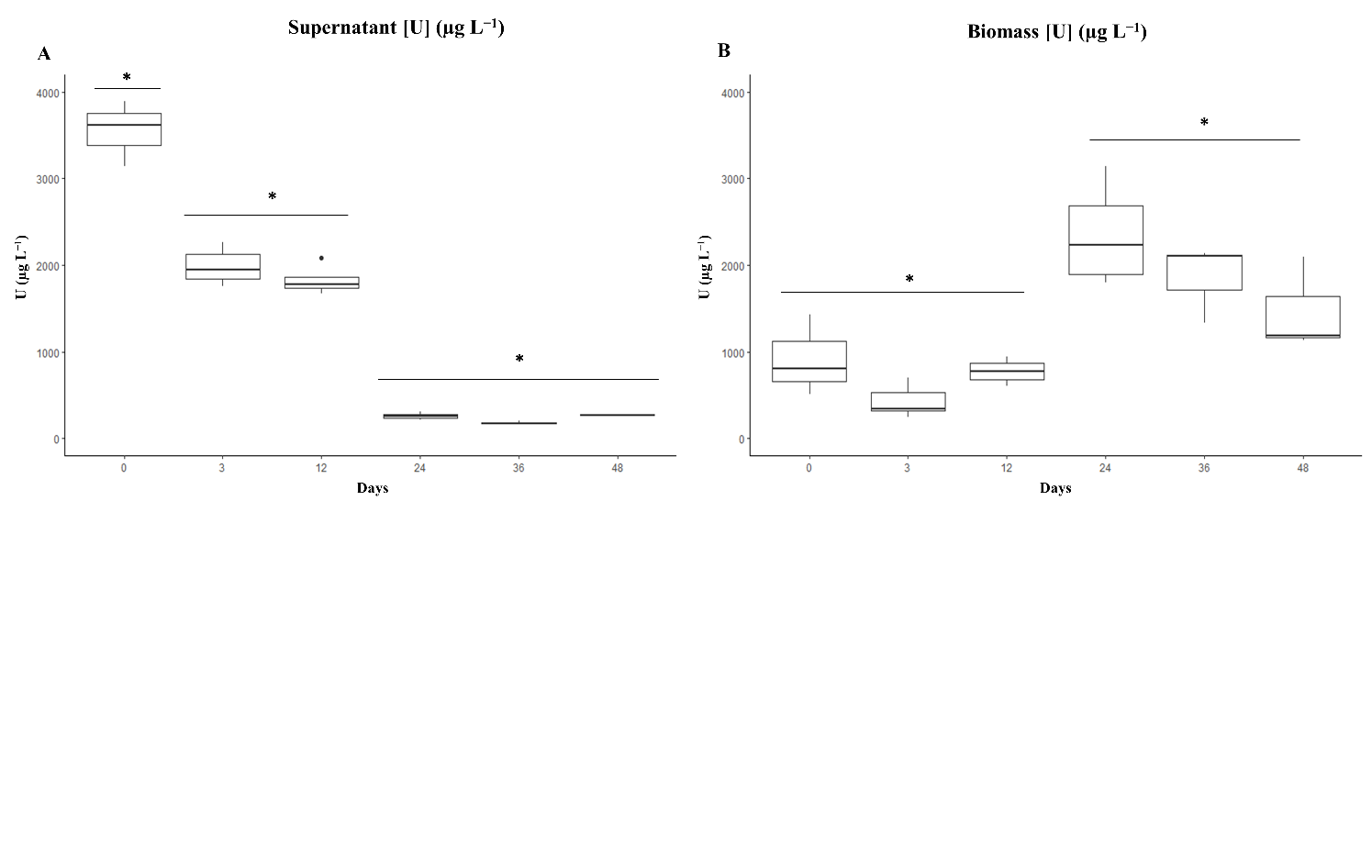


**Supplementary Figure 1.** Boxplots of total U concentration in the supernatant (**A**) and in the ChlSG pellet (**B**) in terms of µg L^−1^ during up to late stationary phase (48 days). * indicates significant differences between boxplots grouped (*P* < 0.05).

# Supplemental videos

## Supplemental Video 1

time-lapse\Video S1. Control time-lapse imagine analysis.AVI

**Video S1.** Time-Lapse imagine video of ChlSG in BG-11 medium, acting as a control. Cells emission values was constant along the experiment after 20h, displaying a red-fluoresce characteristic of the maximum emission of chlorophyll *b* at 670 nm.

## Supplemental Video 2

time-lapse\Video S2. U positive time-lapse imagine analysis.AVI

**Video S2.** Time-Lapse imagine video of ChlSG cells in BG-11 medium enriched with 25 mg L^−1^ of U, in vivo visualization of the U-uptake process. Cells emission values varied along the experiment, the red-fluoresce initially emitted by the cells was gradually replaced by a blue-fluoresce (U fluorescence signal around 510 nm).

# Supplemental tables

## Supplemental table 1

**Supplemental Table 1.** Summary of microorganism U sorption data from the literature.

| **Biomass** | **U concentration (mg C)*** | **qb (mg g^−1^ DB)*** | **Type of Biomass** | **Immobilization** | **Reference** |
| --- | --- | --- | --- | --- | --- |
| *Arthobacter nicotianae* (1) multi-species | 142 [UO_2_(NO_3_)_2_] | 698 | Alive/ Death | Suspension /Immobilized | (Tsuruta, 2002) |
| *Arthrobacter simplex* (1) | 10 n.d. | 58 | n.d. | n.d. | (Sakaguchi and Nakajima, 1991) |
| Bacterial consortia (1) | 100 [UO_2_(NO_3_)_2_] | 218 ± 2 | Alive | Immobilized | (Nancharaiah et al., 2006) |
| *Myxococcus xanthus* (1) | 0.1-03 mM [UO_2_(NO_3_)_2_]6H_2_O | 2.4 mM | Alive/ Death | Suspension | (González-Muñoz et al., 1997) |
| *Pseudomonas aeruginosa* (1) | 100 [UO_2_(NO_3_)_2_] 6H_2_O | 100 | Alive/ Death | Suspension | (Hu et al., 1996) |
| *Pseudomonas aeruginosa* CSU (1) | 100 [UO_2_(NO_3_)_2_] 6H_2_O | 5.36 | Alive | Immobilized | (Hu and Reeves, 1997) |
| *Pseudomonas fluorescens* (1) | 50-1000 [UO_2_(NO_3_)_2_] | 6 | Death | Suspension | (Tsezos and Volesky, 1981) |
| *Pseudomonas saccharophilia* (1) | 10 n.d. | 87 | n.d. | n.d. | (Sakaguchi and Nakajima, 1991) |
| *Pseudomonas sp.* (1) | 100 [UO_2_(NO_3_)_2_] | 202 | Death | Immobilized | (D’Souza et al., 2006) |
| *Pseudomonas sp.* EPS-5028 (1) | 5-500 [UO_2_(NO_3_)_2_] 6H_2_O | 55 | Alive | Suspension | (Marqués et al., 1991) |
| *Pseudomonas sp.(*1) | 100 [UO_2_(NO_3_)_2_] | 541 | Alive/ Death | Suspension/ Immobilized | (Sar et al., 2004) |
| *Streptomyces albus* (1) | 5-10 M [UO_2_(NO_3_)_2_] | 52-54 | Alive | Immobilized | (Nakajima and Sakaguchi, 1986) |
| *Streptomyces longwoodensis* (1) | 5-250 [UO_2_(NO_3_)_2_] | 450 | Alive | Suspension | (Friis and Myers-Keith, 1986) |
| *Streptomyces niveus* (1) | 50-1000 [UO_2_(NO_3_)_2_] | 40 | Death | Suspension | (Tsezos and Volesky, 1981) |
| *Aspergillus flavus* (2) | 1mM [UO_2_(NO_3_)_2_] | 40 | Death | Suspension | (Hafez et al., 1997) |
| *Aspergillus fumigatus* (2) | 200-1000 [UO_2_(NO_3_)_2_] 6H_2_O | 170 | Death | Suspension | (Bhainsa and D’Souza, 1999) |
| *Aspergillus niger* (2) | 10 n.d. | 29 | n.d. | n.d. | (Sakaguchi and Nakajima, 1991) |
| *Aspergillus niger* (2) multi-species | 8.5-1000 | 43 | Death | Suspension | (Kuyucak and Volesky, 1988) |
| *Coniochaeta fodinicola* (2) | 0.5 mM | 43 | Death | Suspension | (Vázquez-Campos et al., 2015) |
| *Penicillium citrinum* (2) | 50 U_3_O_8_ | 127.3 | Death | Suspension | (Pang et al., 2011) |
| *Penicillium spp.* (2) | n.d. | 20.3 | n.d. | n.d. | (McCready and Lakshamanan, 1986) |
| *Phanerochaete chrysosporium* (2) | 100–1000 | 158.0 | Death | Immobilized | (Genç et al., 2003) |
| *Rhizopus* (2) | 200-1000 [UO_2_(NO_3_)_2_] 6H_2_O | 180 | Death | Suspension | (Bhainsa and D’Souza, 1999) |
| *Rhizopus arrhizus* (2) | 100 [U (VI)] | 160 | Death | Suspension | (Tsezos and Volesky, 1982) |
| *Rhizopus arrhizus* (2) | 50-1000 UO_2_(NO_3_)_2_] | 180 | Death | Suspension | (Tsezos and Volesky, 1981) |
| *Rhizopus arrhizus* (2) | 200-500 U mineral | 50 | Death | Immobilized | (Tsezos et al., 1989) |
| *Rhizopus arrhizus* (2) | 10 n.d. | 34 | n.d. | n.d. | (Sakaguchi and Nakajima, 1991) |
| *Rhizopus arrhizus* (2) | n.d. | 42.3 | n.d. | n.d. | (McCready and Lakshamanan, 1986) |
| *Rhizopus arrhizus* chitin (2) | 100 [UO_2_(NO_3_)_2_] | 100 | Death | Suspension | (Tsezos, 1983) |
| *Talaromyces emersonii* CBS 814.70 (2) | 15-300 [UO_2_(CH_3_COO)_2_] H_2_O_2_ | 280 | Alive | Suspension | (Bengtsson et al., 1995) |
| *Trametes versicolor* (2) | 100–1000 | 309.1 | Death | Immobilized | (Genç et al., 2003) |
| *Trichoderma harzianu* (2) | 1000 [UO_2_(CH_3_COO)_2_]_2_ H_2_O_2_ | 612 | Alive/ Death | Suspension | (Akhtar et al., 2007) |
| *Trichoderma harzianum* (2) | 100-1000 [UO_2_(CH_3_COO)_2_]_2_ H_2_O_2_ | 80-95% elimination | Alive | Immobilized | (Akhtar et al., 2009) |
| Beer yeasts after used (3) | 1mM [UO_2_(CH_3_COO)_2_]_2_ H_2_O_2_ | 360 | Death | Suspension | (Riordan et al., 1997) |
| *Kluyveromyces marxianus* IMB3 (3) | 5-100 [UO_2_(CH_3_COO)_2_]_2_ H_2_O_2_ | 115 | Death | Suspension | (Bustard et al., 1996) |
| *Saccharomyces cerevisiae* (3) | 0,1-0,5 mmol L**^−1^** [UO_2_(NO_3_)_2_] 6H_2_O | 2-4 mmol U/g | Death | Suspension | (Omar et al., 1996) |
| *Saccharomyces cerevisiae* (3) | 10 n.d. | 12 | n.d. | n.d. | (Sakaguchi and Nakajima, 1991) |
| *Saccharomyces cerevisiae* (3) multi-species | 8.5-1000 | 157 | Death | Suspension | (Kuyucak and Volesky, 1988) |
| Whisky distillation biomass (3) | 0.1-2 mM [UO_2_(CH_3_COO)_2_]_2_ H_2_O_2_ | 160–170 | Death | Immobilized | (Bustard and McHale, 1997) |
| *Chlorella* (4) | n.d. | 28.5 | n.d. | n.d. | (Matson et al., 2006) |
| *Chlorella regularis* (4) | 1 n.d. | 3.96 | n.d. | n.d. | (Sakaguchi and Nakajima, 1991) |
| *Chlorella regularis* (4) | 2-10 [UO_2_(CH_3_COO)_2_]_2_ H_2_O_2_ | 15.6 | Alive/ Death | Suspension | (Horikoshi et al., 1979) |
| *Chlorella vulgaris* (4) | 0.005-1 mM | 28.3 ± 0.6 | Alive/ Death | Suspension | (Vogel et al., 2010) |
| Microalgae strains RD256 y RD257 (4) | 1000 [UO_2_(CH_3_COO)_2_]_2_ H_2_O_2_ | 354- 408 | Alive/ Death | Suspension | (Akhtar et al., 2007) |
| *Scenedesmus obliquus* 34 (4) | 5,5 [UO_2_(CO_3_)_2_] | 75 | Alive | Suspension | (Zhang et al., 1997) |
| *Scenedesmus sp.* (4) | n.d. | 40.7 | n.d. | n.d. | (Li et al., 2016) |
| *Streptomyces sp.* (4) | 0.7-10 mmol L**^−1^** [UO_2_(CO_3_)_2_] | 150 | Alive/ Death | Suspension | (Golab et al., 1991) |
| *Anacystis nidulans* (5) | 170.8 ±3,4 [UO_2_(CO_3_)_2_] | 0,85 | Death | Suspension | (Liu and Wu, 1993) |
| Cyanobacteria bloom, *Microcystis aeruginosa* (5) | 40-600 [UO_2_(NO_3_)_2_] 6H_2_O | 180 | Death | Suspension | (Li et al., 2004) |
| *Synechococcus elongatus* BDU/75042 (5) | 1M [UO_2_(CO_3_)_2_] | 53.5 | Alive/ Death | Suspension | (Acharya et al., 2009) |

^a^ 1 = bacterium; 2 = fungus; 3 = yeast; 4 = microalgae; 5 = cyanobacteria.

^b^ Highest U uptake value observed.

^*^ Concentration units unless otherwise indicated.

n.d. not determinated.

# References

Acharya, C., Joseph, D., and Apte, S. K. (2009). Uranium sequestration by a marine cyanobacterium, *Synechococcus elongatus* strain BDU/75042. *Bioresour. Technol.* 100, 2176–2181. doi:10.1016/j.biortech.2008.10.047.

Akhtar, K., Khalid, A. M., Akhtar, M. W., and Ghauri, M. A. (2009). Removal and recovery of uranium from aqueous solutions by Ca-alginate immobilized *Trichoderma harzianum*. *Bioresour. Technol.* 100, 4551–4558. doi:10.1016/j.biortech.2009.03.073.

Akhtar, K., Waheed Akhtar, M., and Khalid, A. M. (2007). Removal and recovery of uranium from aqueous solutions by *Trichoderma harzianum*. *Water Res.* 41, 1366–1378. doi:10.1016/j.watres.2006.12.009.

Bengtsson, L., Johansson, B., Hackett, T. J., McHale, L., and McHale, A. P. (1995). Studies on the biosorption of uranium by *Talaromyces emersonii* CBS 814.70 biomass. *Appl. Microbiol. Biotechnol.* 42, 807–11.

Bhainsa, K. C., and D’Souza, S. F. (1999). Biosorption of uranium(VI) by *Aspergillus fumigatus*. *Biotechnol. Tech.* 13, 695–699. doi:10.1023/A:1008915814139.

Bustard, M., Donnellan, N., Rollan, A., McHale, L., and McHale, A. P. (1996). The effect of pulse field strength on electric field stimulated biosorption of uranium by *Kluyveromyces marxianus* IMB3. *Biotechnol. Lett.* 18, 479–482. doi:10.1007/BF00143474.

Bustard, M., and McHale, A. P. (1997). Biosorption of uranium by cross-linked and alginate immobilized residual biomass from distillery spent wash. *Bioprocess Eng.* 17, 127–130. doi:10.1007/s004490050365.

D’Souza, S. F., Sar, P., Kazy, S. K., and Kubal, B. S. (2006). Uranium Sorption by *Pseudomonas* Biomass Immobilized in Radiation Polymerized Polyacrylamide Bio-Beads. *J. Environ. Sci. Heal. Part A* 41, 487–500. doi:10.1080/10934520500428377.

Friis, N., and Myers-Keith, P. (1986). Biosorption of uranium and lead by *Streptomyces longwoodensis*. *Biotechnol. Bioeng.* 28, 21–28. doi:10.1002/bit.260280105.

Genç, Ö., Yalçınkaya, Y., Büyüktuncel, E., Denizli, A., Arıca, M. ., and Bektaş, S. (2003). Uranium recovery by immobilized and dried powdered biomass: characterization and comparison. *Int. J. Miner. Process.* 68, 93–107. doi:10.1016/S0301-7516(02)00062-5.

Golab, Z., Orlowska, B., and Smith, R. W. (1991). Biosorption of lead and uranium by *Streptomyces sp.* *Water. Air. Soil Pollut.* 60, 99–106. doi:10.1007/BF00293968.

González-Muñoz, M. T., Merroun, M. L., Ben Omar, N., and Arias, J. M. (1997). Biosorption of uranium by *Myxococcus xanthus.* *Int. Biodeterior. Biodegradation* 40, 107–114. doi:10.1016/S0964-8305(97)00041-3

Hafez, N., Abdel-Razek, A. S., and Hafez, M. B. (1997). Accumulation of Some Heavy Metals on *Aspergillus flavus*. *J. Chem. Technol. Biotechnol.* 68, 19–22. doi:10.1002/(SICI)1097-4660(199701)68:1<19::AID-JCTB508>3.0.CO;2-K.

Horikoshi, T., Nakajima, A., and Sakaguchi, T. (1979). Uptake of Uranium by *Chlorella regularis*. *Agric. Biol. Chem.* 43, 617–623. doi:10.1080/00021369.1979.10863502.

Hu, M. Z.-C., Norman, J. M., Faison, B. D., and Reeves, M. E. (1996). Biosorption of uranium by *Pseudomonas aeruginosa* strain CSU: Characterization and comparison studies. *Biotechnol. Bioeng.* 51, 237–247. doi:10.1002/(SICI)1097-0290(19960720)51:2<237::AID-BIT14>3.0.CO;2-J.

Hu, M. Z. C., and Reeves, M. (1997). Biosorption of Uranium by *Pseudomonas aeruginosa* Strain CSU Immobilized in a Novel Matrix. *Biotechnol. Prog.* 13, 60–70. doi:10.1021/bp9600849.

Kuyucak, N., and Volesky, B. (1988). Biosorbents for recovery of metals from industrial solutions. *Biotechnol. Lett.* 10, 137–142. doi:10.1007/BF01024641.

Li, P.-F., Mao, Z.-Y., Rao, X.-J., Wang, X.-M., Min, M.-Z., Qiu, L.-W., et al. (2004). Biosorption of uranium by lake-harvested biomass from a cyanobacterium bloom. *Bioresour. Technol.* 94, 193–195. doi:10.1016/j.biortech.2003.11.024.

Li, X., Hu, H., Yu, J., and Zhao, W. (2016). Selection of Suitable Microalgal Species for Sorption of Uranium in Radioactive Wastewater Treatment. *Huan Jing Ke Xue* 37, 1858–63.

Liu, H., and Wu, J. (1993). Uptake and recovery of americium and uranium by *Anacystis* biomass. *J. Environ. Sci. Health . Part A Environ. Sci. Eng. Toxicol.* 28, 491–504. doi:10.1080/10934529309375890.

Marqués, A., Roca, X., Simon-Pujol, M. D., Fuste, M. C., and Congregado, F. (1991). Uranium accumulation by *Pseudomonas sp.* EPS-5028. *Appl. Microbiol. Biotechnol.* 35, 406–410. doi:10.1007/BF00172734.

Matson, C. W., Lambert, M. M., McDonald, T. J., Autenrieth, R. L., Donnelly, K. C., Islamzadeh, A., et al. (2006). Evolutionary toxicology: population-level effects of chronic contaminant exposure on the marsh frogs (*Rana ridibunda*) of Azerbaijan. *Environ. Health Perspect.* 114, 547–52. doi: 10.1289/ehp.8404.

McCready, R. G. L., and Lakshamanan, V. I. (1986). “Review of bioadsorption research to recover uranium from leach solutions in Canada,” in *Immobilisation of ions by bio-sorption*, ed. S. Eccles (Ottawa, Canada), 219–225.

Nakajima, A., and Sakaguchi, T. (1986). Selective accumulation of heavy metals by microorganisms. *Appl. Microbiol. Biotechnol.* 24, 59–64. doi:10.1007/BF00266286.

Nancharaiah, Y. V., Joshi, H. M., Mohan, T. V. K., Venugopalan, V. P., and Narasimhan, S. V.  (2006). Aerobic granular biomass: a novel biomaterial for efficient uranium removal. *Curr. Sci.* 91, 5503–509. doi: 10.1016/j.biortech.2017.09.131.

Omar, N. B., Merroun, M. L., González-Muñoz, M. T., and Arias, J. M. (1996). Brewery yeast as a biosorbent for uranium. *J. Appl. Bacteriol.* 81, 283–287. doi:10.1111/j.1365-2672.1996.tb04330.x.

Pang, C., Liu, Y.-H., Cao, X.-H., Li, M., Huang, G.-L., Hua, R., et al. (2011). Biosorption of uranium(VI) from aqueous solution by dead fungal biomass of *Penicillium citrinum*. *Chem. Eng. J.* 170, 1–6. doi:10.1016/j.cej.2010.10.068.

Riordan, C., Bustard, M., Putt, R., and McHale, A. P. (1997). Removal of uranium from solution using residual brewery yeast: combined biosorption and precipitation. *Biotechnol. Lett.* 19, 385–388. doi:10.1023/A:1018367304767.

Sakaguchi, T., and Nakajima, A. (1991). “Accumulation of heavy metals such as uranium and thorium by microorganisms,” in *Mineral Bioprocessing*, eds. R. W. Smith and M. Misra (The Minerals, Metals and Materials Society), 309-322.

Sar, P., K. Kazy, S., and D’Souza, S. F. (2004). Radionuclide remediation using a bacterial biosorbent. *Int. Biodeterior. Biodegradation* 54, 193–202. doi:10.1016/j.ibiod.2004.05.004.

Tsezos, M. (1983). The role of chitin in uranium adsorption by *R. arrhizus*. *Biotechnol. Bioeng.* 25, 2025–40. doi:10.1002/bit.260250812.

Tsezos, M., McCready, R. G., and Bell, J. P. (1989). The continuous recovery of uranium from biologically leached solutions using immobilized biomass. *Biotechnol. Bioeng.* 34, 10–7. doi:10.1002/bit.260340103.

Tsezos, M., and Volesky, B. (1981). Biosorption of uranium and thorium. *Biotechnol. Bioeng.* 23, 583–604. doi:10.1002/bit.260230309.

Tsezos, M., and Volesky, B. (1982). The mechanism of uranium biosorption by  *Rhizopus arrhizus*. *Biotechnol. Bioeng.* 24, 385–401. doi:10.1002/bit.260240211.

Tsuruta, T. (2002). Removal and recovery of uranyl ion using various microorganisms. *J. Biosci. Bioeng.* 94, 23–28. doi:10.1016/S1389-1723(02)80111-6.

Vázquez-Campos, X., Kinsela, A. S., Collins, R. N., Neilan, B. A., Aoyagi, N., and Waite, T. D. (2015). Uranium Binding Mechanisms of the Acid-Tolerant Fungus *Coniochaeta fodinicola*. *Environ. Sci. Technol.* 49, 8487–8496. doi:10.1021/acs.est.5b01342.

Vogel, M., Günther, A., Rossberg, A., Li, B., Bernhard, G., and Raff, J. (2010). Biosorption of U(VI) by the green algae *Chlorella vulgaris* in dependence of pH value and cell activity. *Sci. Total Environ.,* 409(2): 384-395. doi:10.1016/j.scitotenv.2010.10.011.

Zhang, X., Luo, S., Yang, Q., Zhang, H., and Li, J. (1997). Accumulation of uranium at low concentration by the green alga *Scenedesmus obliquus* 34. *J. Appl. Phycol.* 9, 65–71.
